# Safety and immunogenicity of a third-dose homologous BBIBP-CorV boosting vaccination: interim results from a prospective open-label study

**DOI:** 10.1080/22221751.2022.2025746

**Published:** 2022-02-23

**Authors:** Jingwen Ai, Yi Zhang, Haocheng Zhang, Qiran Zhang, Zhangfan Fu, Ke Lin, Jieyu Song, Yuanhan Zhao, Mingxiang Fan, Hongyu Wang, Yang Zhou, Xiaohua Chen, Chao Qiu, Wenhong Zhang

**Affiliations:** a Department of Infectious Diseases, National Medical Center for Infectious Diseases, Shanghai Key Laboratory of Infectious Diseases and Biosafety Emergency Response, Huashan Hospital, Shanghai Medical College, Fudan University, Shanghai, People’s Republic of China; b Department of Infectious Diseases, Shanghai Jiao Tong University Affiliated Sixth People’s Hospital, Shanghai, People’s Republic of China; c National Clinical Research Center for Aging and Medicine, Huashan Hospital, Fudan University, Shanghai, People’s Republic of China; d Key Laboratory of Medical Molecular Virology (MOE/MOH) Shanghai Medical College, Fudan University, Shanghai, People’s Republic of China

**Keywords:** SARS-CoV-2 inactivated vaccines, homologous, booster vaccination shot, immunogenicity, safety

## Abstract

A COVID-19 booster vaccination is being comprehensively evaluated globally due to the emerging concern of reduced protection rate of previous vaccination and circulating Variants of Concern (VOC). But the safety and immunogenicity of homologous BBIBP-CorV boosting vaccination are yet to be thoroughly evaluated. We conducted this prospective, open-label study in Huashan Hospital using a third 6.5U BBIBP-CorV administered at an interval of 4–8 months following the previous two doses in healthy adults. Safety, anti-RBD response and neutralizing titers against SARS-CoV-2 and VOCs were examined. Sixty-three and forty participants entered the booster and the control group, respectively. A significant increase in IFN-γ SFU per million PBMCs was observed on day 14 against N peptide (20 vs. 5, *P *< 0.001). On day 14, pVNT GMTs increased over 15 folds of the baseline levels against prototype to reach 404.54 titers and over 9–13 folds against 4 VOCs and continuously increased by day 28. sVNT GMTs increased 112.51 and 127.45 folds by days 14 and 28 compared to the baseline level. Median anti-RBD antibody and IgG level significantly increased from 11.12 to 2607.50 BAU/ml and 4.07 to 619.20 BAU/ml on day 14. On day 14, females showed a significantly higher cell-mediated immune response against S1 peptide. The 7–8 months interval group had a higher humoral response than the 4–6 months interval group. No severe adverse event was reported. A third homologous BBIBP-CorV boosting vaccination was safe and highly immunogenic for healthy adults and broadened participants’ immunity against VOCs.

## Introduction

Coronavirus Disease 2019 (COVID-19), caused by SARS-CoV-2, has led to around 250 million infections by October 2021. Due to transmission and adaptations, SARS-CoV-2 variants continued to emerge and circulate globally. Variants of concern (VOC) and variants of interest (VOI) strains changed the virus characteristics and aroused great concerns globally. As of September 2021, more than 6 billion vaccine doses have been administered globally, equalling 90 doses injected for every 100 people. The vaccination percentage in China has reached 161 doses per 100 people, and more than 76% were fully vaccinated [1].

Despite high COVID-19 vaccine coverage, the waning of the elicited immune response has aroused great concern. The neutralizing antibodies generated from two doses of CoronaVac vaccine revealed a worrying trend, with average titer decreasing to 1:8 after 6 months post the second vaccination [2,3]. mRNA vaccine effectiveness against infection among health care personnel peaked on 3–4 weeks after the second dose (96.3%)[4] and dropped to approximately 65% at 6 months post the second dose with a continuous declination. In Qatar, the protection against infections was 77.5% at one month after the second dose of BNT162b2, and only 17.3% to 22.5% at 5–7 months post previous vaccination [5].

With the concern of waning immunity and circulating SARS-CoV-2 variants, booster vaccination has been promoted. On 22nd September, the USA Food and Drug Administration authorized BNT162b2 booster shots for previously vaccinated people aged 65 and older [6]. World Health Organization advised that an additional (third) homologous dose of the Sinovac and Sinopharm inactivated virus vaccines should be offered to those over 60 years old in October 2021[7]. Israel started the third-dose vaccination of BNT162b2 mRNA vaccine as a booster shot on 30 July 2021. Up to now, more than 40% of Israel’s population has been administrated booster after “priming” shots.

The safety and immunogenicity of booster COIVD-19 vaccinations have been tested in various situations with various vaccines. A third homologous boosting shot of inactivated CoronaVac, BBIBP-CorV, BNT162b2, and recombinant adenovirus type-5 (Ad5)-vectored vaccination all had a favourable safety profile and significantly improved immune response[2,8,9]. Heterologous booster immunization was safe and well-tolerated as homologous vaccination, BNT162b2 and Ad5-vectored vaccine booster in participants, who previously received 2-dose inactivated vaccines, could lead to significantly increased viral neutralizing antibodies [10,11]. In Chile, following previous priming vaccination by two doses of CoronaVac, effectiveness against infection of a third booster shot of CoronaVac, BNT162b2, and AZD1222 at 14 days post-vaccination was 56–80%, 56–90%, and 56–93%, respectively[12].

Up until now, different strategies for prime-boost vaccination are still being explored globally, but the safety and immunogenicity of BBIBP-CorV boosting vaccination are yet to be thoroughly evaluated. Therefore, we conducted this prospective, open-label trial to further analyse the immune response and reactogenicity of a third-dose homologous BBIBP-CorV boosting vaccination.

## Materials and methods

### Study design and participants

We conducted this prospective, open-label study in a single centre (Huashan Hospital, National Medical Center for Infectious Diseases [NMCID], Shanghai, China) to explore the safety and immunogenicity of a third homologous boosting vaccination using 6.5U of inactivated SARS-CoV-2 vaccine (BBIBP-CorV) administered at an interval of 4–8 months, following previous two doses of BBIBP-CorV shots in healthy adults aged 18–59 years. The detailed inclusion and exclusion criteria are listed in Supplementary Table 1. Participants entered the booster group or the control group non-randomly after screening for eligibility. Participants in the booster group received a booster vaccination shot at baseline, while participants in the control group received no intervention. Written informed consent was obtained before the enrolment. The study protocol and informed consent form were approved by Huashan Hospital Ethics Committees (Ethical number: KY2021-749). This study was registered at ClinicalTrials.gov, NCT05095298.

### Detection of SARS-CoV-2-specific cellular immune response

To assess SARS-CoV-2-specific T-cell response, we employed the Human IFN-gamma ELISpot kit (Fosun Pharma, Shanghai, China). Participants’ peripheral blood mononuclear cell (PBMC) samples were taken on Day 0, 14, and 28 from the booster group and Day 0 from the control group. S1, S2, and N peptide pools were used to stimulate isolated PBMC for 20 h at 37°C with 5% CO_2_. As a positive control, phytohemagglutinin was introduced, and cells cultivated without stimulations served as a negative control. Following incubation, the detection antibody was added as directed by the manufacturer. ELISpot Reader version 7.0 (Autoimmun Diagnostika GmbH, Germany) was used to count IFN-producing spots. Then the IFN-γ spot-forming units (SFU) per million PBMC were calculated.

### Detection of anti-SARS-CoV-2 receptor-binding domain (RBD) neutralizing responses, antibody and IgG

We assessed the anti-RBD responses induced by a third boosting vaccination, including plasma surrogate virus neutralization test (sVNT), anti-RBD antibody and IgG test. Blood samples were taken from participants for serology tests at days 0, 14 and 28 after the boosting vaccination. Plasma sVNT titer was determined using a SARS-CoV-2 Neutralizing Ab detection kit (PerkinElmer SuperFlex Anti-SARS-CoV-2 Neutralizing Ab Kit, SDX-57042). The anti-RBD antibody and IgG were measured by a PerkinElmer SuperFlex Anti-SARS-CoV-2 Ab Kit and a SuperFlex Anti-SARS-CoV-2 IgG Kit.

According to the manufactory brochures (www.perkinelmer.com), we used superparamagnetic microparticles and direct chemiluminescence technology to detect antibody in plasma samples. Plasma was serially diluted before detection, 50 μl diluted sample was added to sample wells and then mixed with 50 ul SARS-CoV-2 receptor-binding domain protein labelled with acridinium ester. Signals were captured using PerkinElmer SuperFlex automatic chemiluminescence immunoassay analyser.

To measure the neutralizing titer, the signals were converted to sVNT titer using a reference standard curve plotted with kit-suppled reagents. The sVNT titer was determined by the reciprocal of the last dilution resulting in >50% reduction of chemiluminescence signal. The concentration of the anti-SARS-CoV-2 antibody or IgG of the samples was correlated with the luminous intensity. The antibody assay was analysed in its original scale, and results were then converted to the WHO international standard units using the conversion factors supplied by the laboratory.

### Plasma pseudovirus neutralization test (pVNT)

Blood samples were taken from participants of booster groups on days 0, 14 and 28 after the boosting vaccination. The samples were taken from the participants of the control groups on day 0. Pseudovirus incorporating spike protein from prototype or variants (Alpha, Beta, Gamma and Delta) was constructed using the procedure described by Nie et al. [13]. On the day before transfection, 293T cells were prepared and adjusted to the concentration of 5–7 × 10^5^cell/mL with DMEM complete medium. 30 μg of plasmid pcDNA3.1. S2, expressing the spike protein was transfected according to the instruction. Afterwards, diluted G*ΔG-VSV (VSV G pseudovirus) was added into flasks. Serial dilutions of human plasma and pseudoviruses with a concentration of 1300 TCID50/mL were added into the plates. After incubation, HuH-7 cells were added to the plates. Chemiluminescence was detected after 24-hour incubation. A serial fold of dilutions was made, and the last column was used as the cell’s control without pseudovirus. Positive was determined to be ten-fold higher than the negative (cells only) in terms of relative luminescence unit (RLU) values. The Reed-Muench method was used to calculate the virus neutralization titer. The results are based on 3–5 replicates unless specified.

### Assessment of safety

Solicited systemic and local adverse reactions were recorded by the participants on designed electronic questionnaires. Unsolicited adverse reactions were described by participants on electronic questionnaires with open-ended questions with the instructions from site personnel. Electronic questionnaires were distributed and collected by trained site personnel on days 3, day 14 and day 28 after the boosting dose.

### Statistical analysis

We present summary statistics for individuals as median with inter-quartile ranges (IQRs), or geometric mean with 95% confidence intervals (CIs). Adverse reactions post-vaccination was expressed as numbers and proportions. Mann–Whitney U test, Student’s *t*-test or One-way ANOVA test was used for continuous variables, and Pearson χ² test or Fisher’s exact test for categorical variables to assess the statistical significance between groups and subgroups. When comparing immunogenic outcomes, we adjusted for factors that were considered potentially related to immune response according to the previous studies (including gender, age and intervals between the second and third doses), using multiple linear regressions. Hypothesis testing was two-sided and *P* values of less than 0.05 were considered significant. IBM SPSS (version 20.0) and Graphpad Prism (version 9.2) were used for statistical analysis.

## Result

### Study design and participant characteristics

**Figure 1.**
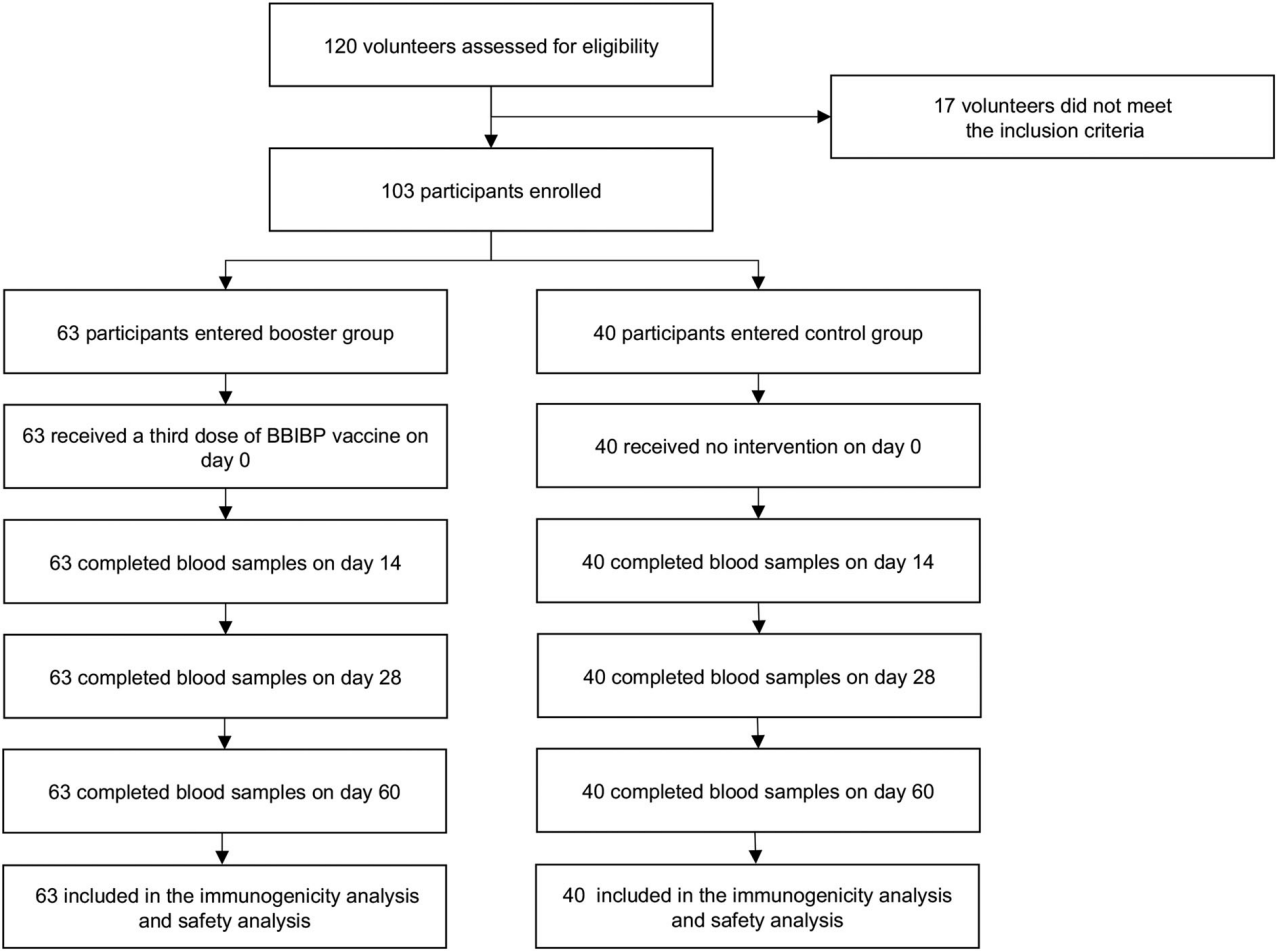
The flowchart of the study.

Between 6 August 6 and 10 October 2021, 120 volunteers were recruited and screened for eligibility (Figure 1). A total of 103 participants were enrolled in the study, of which 63 entered the booster group, and 40 entered the control group. The median age was 28.0 (IQR, 25.0–38.0) in the booster group and 25.0 (24.0–27.8) in the control group (*P *= 0.002). Twenty-seven (42.9%) participants in the booster group and 16 (40.0%) in the control group were males (*P *= 0.774). Body mass index was similar between the booster group and the control group (22.2 vs. 23.2, *P *= 0.400). There was a statistical difference between the booster and the control group concerning intervals between the second and third doses (*P *= 0.033). After stratified with age, all characteristics were similar between the two groups, except for the intervals between the second and third doses. The demographic characteristics are detailed in Table 1.

**Table 1.** Basic characteristics of participants.

	Booster group (*n* = 63)	Control group (*n* = 40)	*P*-value
*Age (years)*	28.0 (25.0–38.0)	25.0 (24.0–27.8)	**0**.**002**
*Sex*			
Male (%)	27 (42.9%)	16 (40.0%)	0.774
Female (%)	36 (57.1%)	24 (60.0%)	
*Interval between the second and third doses*			
4–6 months (%)	29 (46.0%)	27 (67.5%)	**0**.**033**
7–8months (%)	34 (54.0%)	13 (32.5%)	
*BMI*	22.2 (20.4-23.9)	23.2 (20.7-25.4)	0.400
*Ethnic group, Asian*	63 (100.0%)	40 (100.0%)	–
*Age groups*			
<40 years	50/63 (79.4%)	32/40 (80.0%)	0.938
Male (%)	21/50 (42.0%)	13/32 (40.6%)	0.902
Female (%)	29/50 (58.0%)	19/32 (59.4%)	
An interval of 4–6 months between the second and third doses	26/50 (52.0%)	26/32 (81.3%)	**0**.**007**
An interval of 7–8 months between the second and third doses	24/50 (48.0%)	6/32 (18.8%)	
BMI	22.0 (20.4–23.8)	22.4 (20.2–25.3)	0.497
≥40 years	13/63 (20.6%)	8/40 (20.0%)	0.938
Male (%)	6/13 (46.2%)	3/8 (37.5%)	0.999
Female (%)	7/13 (53.8%)	5/8 (62.5%)	
An interval of 4–6 months between the second and third doses	3/13 (23.1%)	1/8 (12.5%)	0.502
An interval of 7–8 months between the second and third doses	10/13 (76.9%)	7/8 (87.5%)	
BMI	22.9 (21.3–24.0)	24.7 (23.5–27.9)	0.080

Note: Date presented as Median (interquartile) or number (percentage).

For all categorical variables, the Chi-Square statistic was used.

Continuous variables were compared using a Mann–Whitney test.

*P* < 0.05 was considered statistically significant for all analyses. BMI = Body Mass Index.

### Baseline T and B cell response to SARS-CoV-2

After 4–8 months of two-dose inactivated vaccinations, individuals had 2.5 (IQR, 0–10), 0 (0–5), and 15(5–35) spots of IFN-SFU per million PBMC against S1, S2, and N peptide in the control group, and 5 (0–15), 5 (0–15), and 5 (0–15) spots in the booster group (Supplementary Figure 1a).

**Figure 2.**
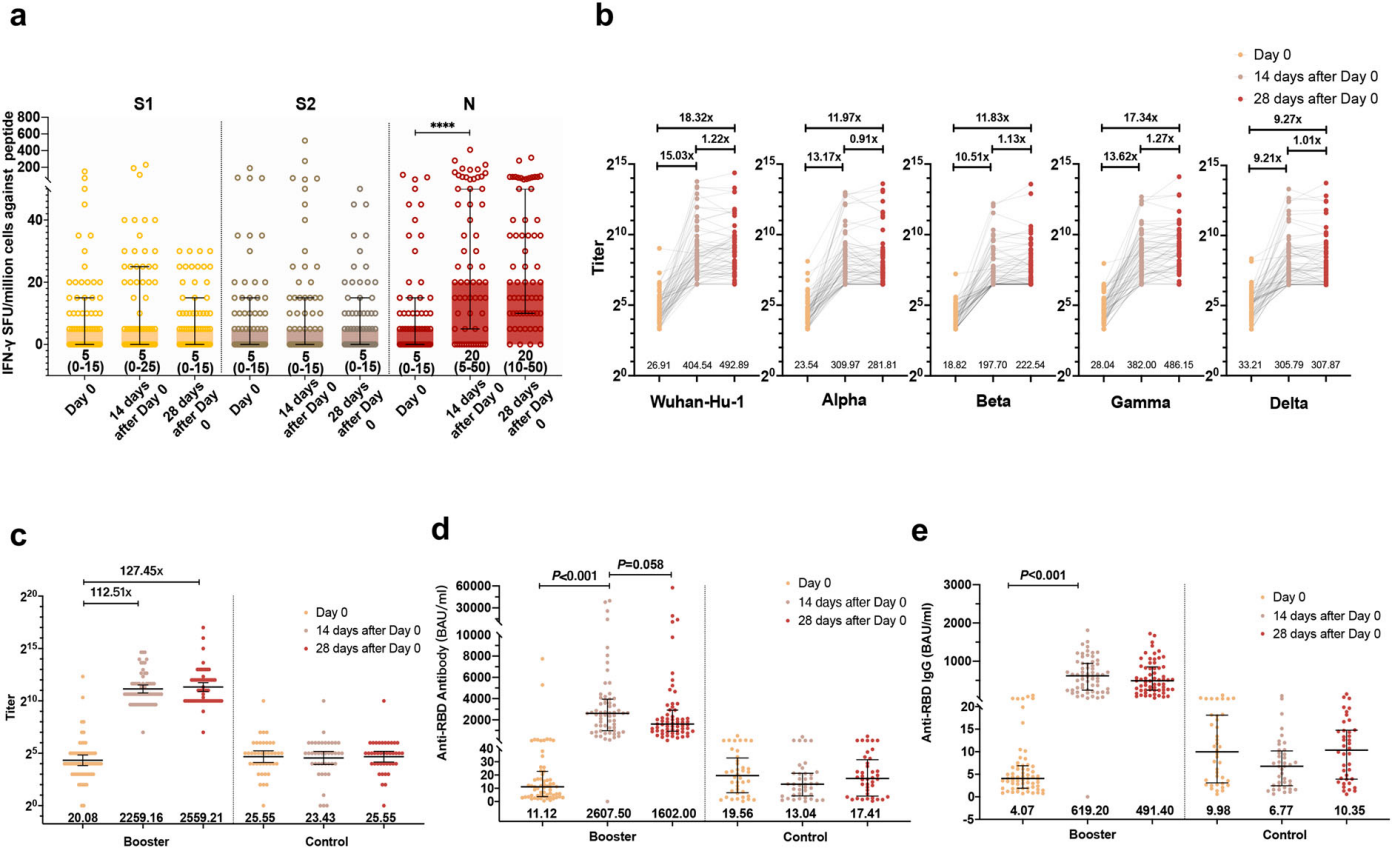
Immune response after the third-dose vaccination. (a) IFN-γ SFU/million PBMCs after the third-dose vaccination. (b) Humoral immune responses against prototype and variants of SARS-CoV-2 after the third-dose vaccination evaluated by pVNT. (c) Humoral immune responses after the third-dose vaccination evaluated by sVNT. (d) Humoral immune responses after the third-dose vaccination evaluated by an anti-RBD antibody. (e) Humoral immune responses after the third-dose vaccination evaluated by anti-RBD IgG.

For the baseline anti-RBD, antibody and neutralizing antibody level, the median of anti-RBD antibody and IgG level was 19.56 (IQR, 6.57–32.87) and 9.98 (3.09–18.10) BAU/mL in the control group, and 11.12 (3.72–22.81) and 4.07(1.91–6.89) BAU/mL in the booster group (Figure 2). Geometric mean titers (GMTs) of sVNT and pVNT were 25.55 (95% CI,17.41–37.49) and 26.09(20.82,32.69) in the control group, and 20.08 (14.18–28.44) and 26.91 (22.22–32.58) in the booster group. pVNT titer in the control and booster groups was similar among prototype strain and VOCs (Figure 2; Supplementary Figure 1b,1c and 2).

### T and B cell response to SARS-CoV-2 post booster vaccination

On Day 14 post the boosting vaccination, there was a substantial increase in N peptide (20 [IQR 5–50] versus 5 [IQR 0–15], *p *< 0.001), but no notable increase in S1 or S2 peptide pools following booster vaccination. From Day 14 to Day 28, IFN-producing T lymphocytes against S1, S2, and N remained stable (Figure 2(a)).

At day 14 post booster vaccination, GMTs of pVNT increased 15.03 (95% CI, 10.34–21.86) folds (Geometric mean fold ratio [GMFR]) against prototype (GMT, 26.91[22.22–32.58] to 404.54[289.99–564.34]), 13.17(9.38–18.49) folds against Alpha (GMT, 23.54[19.83–27.95] to 309.97[225.66–425.77]), 10.51(7.84–14.08) folds against Beta (GMT, 18.82[16.46–21.52] to 197.70[153.07–255.36]), 13.62(10.02–18.52) folds against Gamma (GMT, 28.04[23.77–33.09] to 382.00[288.35–506.08]), and 9.21(6.52–13.00) folds against Delta (GMT, 33.21[27.79–39.68] to 305.79[224.62–416.28]). At day 28 post booster vaccination, GMTs of pVNT further increased to 492.89(359.58–675.62), 281.81(204.8–387.77), 222.54(169.69–291.84), 486.15(367.23–643.58), 307.87(225.75–419.87) against prototype, Alpha, Beta, Gamma and Delta, respectively (Figure 2(b)). The neutralizing antibody-positive rates of sVNT and pVNT on Day 0 were 55.56% and 41.27%, respectively. The seroconversion rates of sVNT were 98.41% on day 14 and increased to 100% on day 28, while the seroconversion rates of pVNT against all VOCs was 100% after day 14. We listed the detailed antibody-positive rates and seroconversion rates in the supplementary material (Supplementary Table 2).

By day 14 and day 28, sVNT GMTs robustly increased to 2259.16 (95% CI, 1734.87–2941.91) and 2559.21(1916.9–3416.75), which were 112.51(74.16–170.69), 127.45(82.31–197.36) times of the baseline levels, respectively (Figure 2(c)). Median anti-RBD antibody level and IgG level were dramatically enhanced to 2607.50 (IQR 972.00–3950.00) BAU/mL and 619.20 (245.90–945.10) BAU/mL on day 14, and decreased slightly to 1602.00 (930.30–2922.00) BAU/mL and 491.40 (246.60–851.90) BAU/mL on day 28 (Figure 2(d,e)). The control group showed a stable profile of low immune response and neutralization potency during our follow-up.

### Subgroup analysis of the immune response by age, gender and interval between the second and third doses in the booster group

An Analysis of the antibody response showed that at day 0, and 14 days or 28 days after the third BBIBP-CorV dose, there were no differences in the humoral response to the prototype and four strains by gender and age (over or under 40 years old) (Supplementary Figure 3). At 14 days after the third BBIBP-CorV dose, the number of spots against S1 peptide in the female group was significantly higher than that of the male group (20[IQR 0–30] vs. 5[0–10], adjusted *P* = 0.019). By day 28 post booster vaccination, no difference in cell-mediated immune response was observed between different genders. At day 0, and 14 days or 28 days after the third BBIBP-CorV dose, the cell-mediated immune response against S1, S2 and N did not vary between age groups (over or under 40 years old) (Supplementary Figure 4).

**Figure 3.**
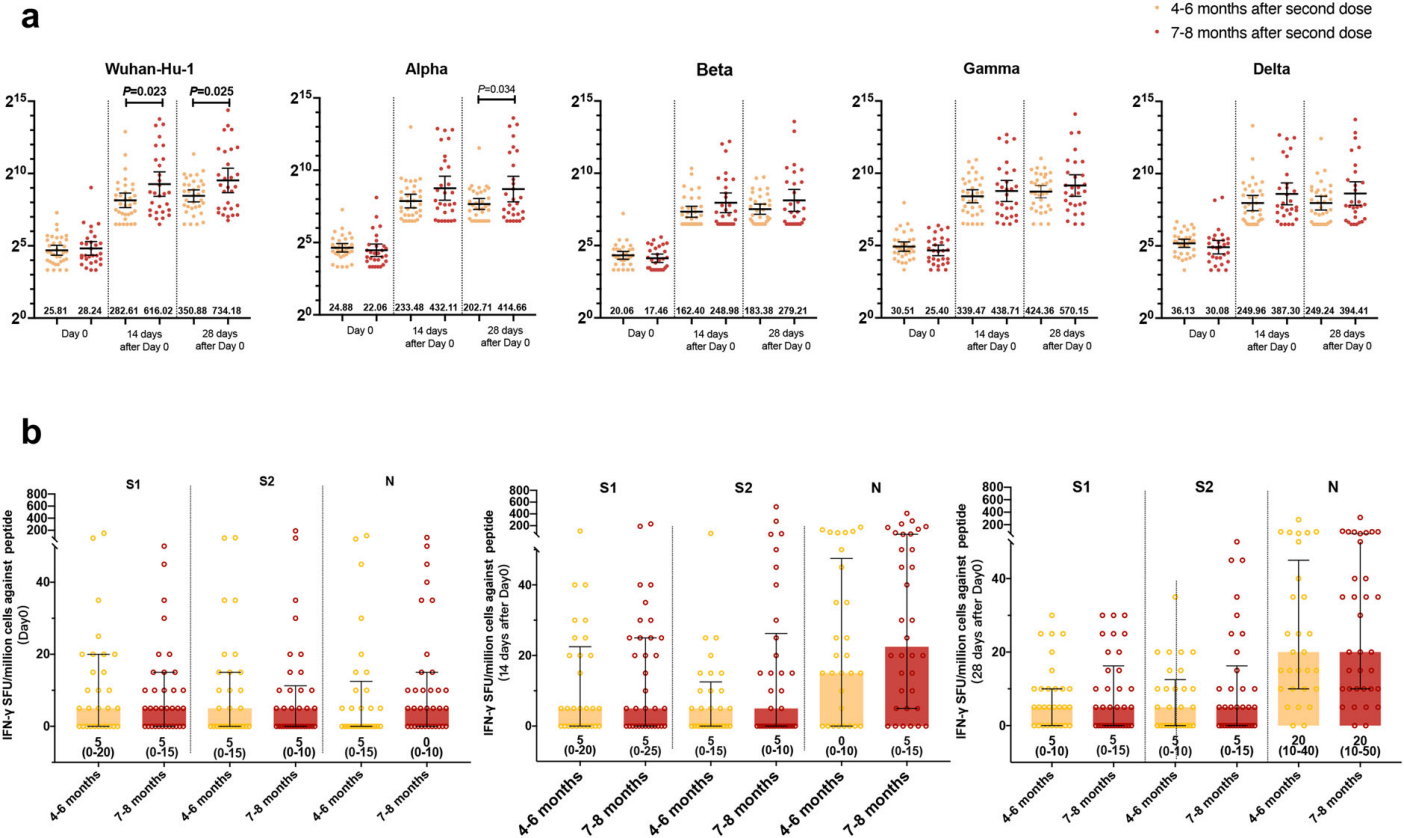
Impact of the interval among three doses of BBIBP-CorV vaccinations on humoral immune responses and T-cell response. (a) pVNT titer against prototype and variants of SARS-CoV-2 in participants administered with third doses at 4–6 months and 7–8 months intervals after second doses; (b) T-cell response in participants administered with third doses at 4–6 months and 7–8 months intervals after second doses.

We further studied the impact of different intervals (4–6 and 7–8 months between the second and third doses) on the host’s immune response after the booster shots (Figure 3). Humoral response analysis showed that at days 14 and 28 post booster vaccination, the 7–8 months interval group had a higher GMTs of pVNT against prototype than the 4–6 months interval group (Day 14, 616.02[95%CI, 343.62–1104.38] vs. 282.61[199.49–400.35], *P *= 0.023; Day 28, 734.18[409.42–1316.53] vs. 350.88[262.76–468.54], *P *= 0.025), while on day 28, the 7–8 months interval group had a higher GMTs of pVNT against Alpha strains than the 4–6 months interval group (414.66[224.26–766.7] vs. 202.71[156.14–263.18], *P *= 0.034). No other difference was observed in antibody response against VOCs and SARS-CoV-2 specific T-cell response by different interval groups (Figure 3(a,b)).

### Safety profile of booster vaccination

In the booster group, solicited injection sites and systemic adverse reactions were reported by 26 (41.3%) and 11 (17.5%) participants within 3 days after the boosting dose. The most common injection site and the systemic adverse reaction were pain (26 [41.3%]) and fatigue (4 [6.3%]). From day 4–14, solicited injection site and systemic adverse reactions were reported by 7 (11.1%) and 2 (3.2%) participants, respectively, of which pain (6[9.5%]) and fever (2 [3.2%]) were the most common reactions. Only 3 (4.8%) and 1 (1.6%) participants reported emerging or persisting solicited injection site or systematic adverse reactions from day 15 to 28, respectively. Unsolicited systemic adverse events were reported by 5 (7.9%) participants from day 0 to 3, 1 (1.6%) from day 4 to 14, and none from day 15 to 28. None of the unsolicited local adverse events was reported (Table 2). The safety data of the control group are displayed in Supplementary table 3.

**Table 2.** Solicited and unsolicited adverse reactions.

	Total*	Day0-Day3	Day4-Day14	Day15-Day28
*Solicited adverse reactions*				
*Injection site adverse reactions*				
Any (%)	28 (44.4%)	26 (41.3%)	7 (11.1%)	3 (4.8%)
Grade 1	28 (44.4%)	26 (41.3%)	7 (11.1%)	3 (4.8%)
Pain	28 (44.4%)	26 (41.3%)	6 (9.5%)	3 (4.8%)
Grade 1	28 (44.4%)	26 (41.3%)	6 (9.5%)	3 (4.8%)
Induration	1 (1.6%)	1 (1.6%)	0 (0.0%)	0 (0.0%)
Grade 1	1 (1.6%)	1 (1.6%)	0 (0.0%)	0 (0.0%)
Swelling	3 (4.8%)	2 (3.2%)	1 (1.6%)	0 (0.0%)
Grade 1	3 (4.8%)	2 (3.2%)	1 (1.6%)	0 (0.0%)
Erythema	0 (0.0%)	0 (0.0%)	0 (0.0%)	0 (0.0%)
Pruritus	0 (0.0%)	0 (0.0%)	0 (0.0%)	0 (0.0%)
*Systematic adverse reactions*				
Any (%)	14 (22.2%)	11 (17.5%)	2 (3.2%)	1 (1.6%)
Grade 1	14 (22.2%)	11 (17.5%)	2 (3.2%)	1 (1.6%)
Fever	3 (4.8%)	2 (3.2%)	2 (3.2%)	0 (0.0%)
Grade 1	3 (4.8%)	2 (3.2%)	2 (3.2%)	0 (0.0%)
Fatigue	5 (7.9%)	4 (6.3%)	0 (0.0%)	1 (1.6%)
Grade 1	5 (7.9%)	4 (6.3%)	0 (0.0%)	1 (1.6%)
Myalgia	2 (3.2%)	2 (3.2%)	0 (0.0%)	0 (0.0%)
Grade 1	2 (3.2%)	2 (3.2%)	0 (0.0%)	0 (0.0%)
Vertigo	3 (4.8%)	3 (4.8%)	0 (0.0%)	0 (0.0%)
Grade 1	3 (4.8%)	3 (4.8%)	0 (0.0%)	0 (0.0%)
Anorexia	1 (1.6%)	0 (0.0%)	0 (0.0%)	1 (1.6%)
Grade 1	1 (1.6%)	0 (0.0%)	0 (0.0%)	1 (1.6%)
Rash	0 (0.0%)	0 (0.0%)	0 (0.0%)	0 (0.0%)
Cough	0 (0.0%)	0 (0.0%)	0 (0.0%)	0 (0.0%)
Arthralgia	0 (0.0%)	0 (0.0%)	0 (0.0%)	0 (0.0%)
Dyspnoea	0 (0.0%)	0 (0.0%)	0 (0.0%)	0 (0.0%)
Nausea	0 (0.0%)	0 (0.0%)	0 (0.0%)	0 (0.0%)
Pharyngalgia	0 (0.0%)	0 (0.0%)	0 (0.0%)	0 (0.0%)
Syncope	0 (0.0%)	0 (0.0%)	0 (0.0%)	0 (0.0%)
Vomiting	0 (0.0%)	0 (0.0%)	0 (0.0%)	0 (0.0%)
*Unsolicited adverse reactions*				
*Injection site adverse reactions*				
Any (%)	0 (0.0%)	0 (0.0%)	0 (0.0%)	0 (0.0%)
*Systematic adverse reactions*				
Any (%)	6 (9.5%)	5 (7.9%)	1 (1.6%)	0 (0.0%)
Grade 1	6 (9.5%)	5 (7.9%)	1 (1.6%)	0 (0.0%)
Diarrhoea	3 (4.8%)	3 (4.8%)	0 (0.0%)	0 (0.0%)
Grade 1	3 (4.8%)	3 (4.8%)	0 (0.0%)	0 (0.0%)
Flatus	1 (1.6%)	1 (1.6%)	0 (0.0%)	0 (0.0%)
Grade 1	1 (1.6%)	1 (1.6%)	0 (0.0%)	0 (0.0%)
Rhinorrhoea	1 (1.6%)	1 (1.6%)	0 (0.0%)	0 (0.0%)
Grade 1	1 (1.6%)	1 (1.6%)	0 (0.0%)	0 (0.0%)
Lethargy	1 (1.6%)	0 (0.0%)	1 (1.6%)	0 (0.0%)
Grade 1	1 (1.6%)	0 (0.0%)	1 (1.6%)	0 (0.0%)

Note: *Total number of participants who had adverse events throughout the 28-day observation. If a participant had a persistent or recurrent symptom, it would be counted only once.

## Discussion

In this study, we administered a prospective, open-label design to assess the immunogenicity and safety of a homologous BBIBP-CorV booster vaccination given four to eight months after the two doses. We found that a third homologous booster dose of BBIBP-CorV was safe and highly immunogenic for healthy adults aged 18–59 years. Previous studies have reported enhanced humoral immune response of the third homologous BBIBP-CorV strategy[9]. But, this study added to the foundational evidence on cell-mediated immune response and immunogenicity against VOCs post boosting vaccination. These findings support the potential use of a third homologous BBIBP-CorV boosting strategy.

Our study showed that two-dose BBIBP-CorV vaccinations are effective in producing functional B and T cell responses, even after 4–8 months, suggesting inactivated vaccines could elicit long-lasting humoral and cell-mediated immunity to protect against SARS-CoV-2 to some degree. As shown in previous studies and our baseline data, two-dose immunization with inactivated vaccines might have more advantage in inducing humoral immune response than cell-mediated immune response [14]. Our results found that the third-dose BBIBP-CorV could recall and quickly elevate the humoral immune response by increasing antibody folds. Other research has reported that a third dose of inactivated SARS-CoV-2 vaccine could further generate SARS-CoV-2-specific CD4 + and CD8+ T cells and elicit B cell response by antibody evolution [15,16].

A few studies have demonstrated the immunogenicity of the third dose homologous and heterologous boosting vaccination. For the homologous studies, the third dose of 30-μg BNT162b2 (mRNA vaccine)[17] found a 5-to 7-fold increase in GMTs, and the people, who received 100-μg mRNA-1273(mRNA vaccine) [18], had a 4-fold increase in the neutralization response. After the ChAdOx1 nCoV-19[19] and Ad26.COV2.S EUA[18] booster (adenovirus vaccine), the GMT level increased to 3.69 and 4.6 times of the baseline, respectively. Previous homologous inactivated vaccine (BBIBP-CorV and CoronaVac) booster study found a 1- to 3-fold increase compared to the GMT level after the second dose [2,9]. A heterologous prime-booster study showed that among participants, received Ad26.COV2.S, mRNA-1273, or BNT162b2 as heterologous booster vaccinations [18], GMT-fold rises in neutralization titers were greatest for Ad26.COV2.S EUA-primed recipients (35.1–75.9 fold), followed by BNT162b2 (12.5–31.7 fold) and mRNA-1273 (6.2–11.5 fold) recipients. While the mRNA-1273 primed participants had the highest GMT level 15 days after the vaccination shots no matter what the booster vaccination they received. According to the existing studies, homologous and heterologous prime-boost strategies may offer immunological advantages to optimize the breadth and longevity of vaccine protection.

Whether enhanced B and T cell responses could lead to higher protection efficacy is one of the key questions concerning the current boosting strategies. A study in Israel reported an 11.3% reduced risk of COVID-19 in the booster group (≥60 years), which received a three-dose BNT162b2 regimen and found that the rate of severe illness was also substantially lower. Such a result supported the concept that a higher immune response would provide better effectiveness in the real world. Another study, among healthcare workers, had shown that an anti-RBD IgG level higher than 506.00 BAU/mL[20] was correlated with a reduced risk of COVID-19 symptomatic infection. This was lower than our peak anti-RBD IgG level of 619.2 BAU/mL 14 days after the booster. Therefore, a third homologous BBIBP-CorV booster could be an efficient strategy to enhance vaccination effectiveness in high-risk groups. However, the antibody level mentioned in the referred article was obtained through modelling using the Alpha variant in a British study. Therefore these results could only partially mirror the possible efficacy of our booster strategy against the Delta variant.

A previous study found that those, who had longer intervals between priming and boosting doses, would have higher antibody levels following the boosting immunization than those who had shorter intervals. In our study, we discovered a similar phenomenon concerning the degree of antibody responses across different intervals (4–6 months and 7–8 months) between the second and third doses[19]. For prototype and Alpha variants, longer intervals (7–8 months) between the second and third BBIBP-CorV might lead to higher neutralization potency, but the most optimized intervals for the boosting vaccination would still rely upon substantial future research.

Our study found that participants older than 40 would have a decreasing trend in humoral immune response compared to those younger than 40. This result is similar to others reported previously and suggested the importance of establishing an optimized boosting strategy in elderly people. We also found that females had a relatively higher T cell immune response than males after booster shots; however, to generalize for a wider population, more data would be needed in the future.

Although a higher incidence rate of injection-site and systemic reactions within 28 days after boost vaccination were reported in this study than the incidences rate previously reported within 28 days after the prime inactivated vaccinations, all adverse symptoms were mild in severity and primarily, transient[21,22]. The reactogenicity profile of the BBIBP-CorV boosting vaccination was similar to that of the previous 2 doses. The results suggest that a third homologous BBIBP-CorV boosting vaccination in healthy adults aged 18–59 years might be safe. These results may help support the booster vaccination strategies to be administered in the future.

Our study has several limitations. Due to the stable immune response during this one month, the limited funding resource and laboratory resource limitation, we only performed pVNT and ELISpot on Day 0 for the control group. Although our study acquired preliminary data, further research is needed before our results can be applied to populations with coexisting chronic diseases or immunocompromised history. What’s more, the efficacy of the vaccines, especially regarding breakthrough SARS-CoV-2 infections, COVID-19 morbidity and mortality following the boosting vaccination, which was not assessed in this study due to the limited cases in China, should further be evaluated in populations with a higher risk of exposure.

## Conclusion

A third homologous BBIBP-CorV boosting vaccination was safe and highly immunogenic for healthy adults, which significantly recalled and increased the body’s immune response and enhanced participants’ immunity against SARS-CoV-2 variants. Prolonged intervals between the second and third doses might further increase the host’s antibody response. Our findings provide an important piece of evidence for establishing a future global homologous boosting strategy against the COVID-19 pandemic.

## Supplementary Material

Supplemental Material
