# Supplementary material for: Safety and immunogenicity of a third-dose homologous BBIBP-CorV boosting vaccination: interim results from a prospective open-label study
Source: Emerg Microbes Infect. 2022 Feb 23;11(1):639–47. doi: 10.1080/22221751.2022.2025746 (PMC8881062; doi:10.1080/22221751.2022.2025746)
Supplement: Supplemental Material [file TEMI_A_2025746_SM1003.docx]

**Supplementary Materials**

**Supplementary Table 1.** Inclusion and Exclusion Criteria in this study

**Supplementary Table 2.** Antibody-positive rates and seroconversion rates

**Supplementary Table 3.** Solicited and unsolicited adverse reactions in control group

**Supplementary Figure 1.** The baseline antibody response and IFN-γ SFU/ million PBMCs 4-8 months after second dose

**Supplementary Figure 2.** Humoral immune responses after the third-dose vaccination evaluated by sVNT(IU/mL) .

**Supplementary Figure 3.** Subgroup analysis of antibody response in boost group evaluated by gender and age (<40 yo, and≥40 yo).

**Supplementary Figure 4.** Subgroup analysis of T-cell response in boost group evaluated by gender and age (<40 yo, and≥40 yo).

**Supplementary Table 1. Inclusion and Exclusion Criteria in this study**

| **Inclusion criteria:** |
| --- |
| 1. Healthy adults, or adults with pre-existing but stable medical conditions (Participants didn’t require significant change in therapy or hospitalization within 3 months before enrollment). |
| 2. Participants who have received priming vaccination by two doses of inactivated whole-virion vaccines (BBIBP-CorV) within 4 to 8 months. |
| 3. Participants that are willingly to comply with the study procedures and provide written informed consent. |
| **Exclusion criteria:** |
| 1. SARS-CoV-2 infection confirmed by positive reverse transcription-polymerase-chain-reaction (RT-PCR) assay. |
| 2. A history of infection with SARS-CoV-2, or a history of contacting with cases of confirmed or suspected SARS-CoV-2 infection. |
| 3. Presence of fever, cough, runny nose, sore throat, diarrhoea, dyspnoea, or tachypnoea within 7 days before screening visit. |
| 4. Allergy to any ingredient included in SARS-CoV-2 vaccines. |
| 5. A history of severe allergy (such as angioneurotic oedema or allergic shock) to any vaccination. |
| 6. A positive blood pregnancy test. |
| 7. A history or family history of mental illness or serious central nervous system diseases (such as epilepsy, transverse myelitis, Guillain Barre syndrome, demyelinating disease, encephalopathy etc.). |
| 8. Suffering from severe liver or kidney disease. |
| 9. Uncontrollable hypertension (systolic blood pressure over 180 mmHg, diastolic blood pressure over 100 mmHg). |
| 10. Diabetes complications. |
| 11. Malignant tumors. |
| 12. Other acute diseases attack or chronic diseases with acute exacerbation. |
| 13. Known history of cancer or solid organ transplant. |
| 14. Known immunosuppressive or immunodeficient state including confirmed HIV infection, and history of receiving systemic immunosuppressants within 3 months prior to the day of screening. |

**Supplementary Table 2.** Antibody-positive rates and seroconversion rates

| **Antibody-positive rates** | **Day 0 (N=63)** | **Day 14 (N=63)** | **Day 28 (N=63)** |
| --- | --- | --- | --- |
| **sVNT** | 35 (55.56%) | 62(98.41%) | 63(100%) |
| **pVNT Wuhan-Hu-1** | 26(41.27%) | 63(100%) | 63(100%) |
| **pVNT Alpha** | 15(23.81%) | 63(100%) | 63(100%) |
| **pVNT Beta** | 12(19.05%) | 63(100%) | 63(100%) |
| **pVNT Gamma** | 25(39.68%) | 63(100%) | 63(100%) |
| **pVNT Delta** | 36(57.14%) | 63(100%) | 63(100%) |

| **Seroconversion rates** | **Day 14-Day 0 (N=63)** | **Day 28-Day 0 (N=63)** |
| --- | --- | --- |
| **sVNT** | 96.42% | 100% |
| **pVNT Wuhan-Hu-1** | 100% | 100% |
| **pVNT Alpha** | 100% | 100% |
| **pVNT Beta** | 100% | 100% |
| **pVNT Gamma** | 100% | 100% |
| **pVNT Delta** | 100% | 100% |

**Supplementary Table 3. Solicited and unsolicited adverse reactions in control group**

|  | **Total (n=40)** | **Day0-Day3** | **Day4-Day14** | **Day15-Day28** |
| --- | --- | --- | --- | --- |
| **Solicited adverse reactions** | |  |  |  |
| **Injection site adverse reactions** | |  |  |  |
| Any (%) | 0 (0.0%) | 0 (0.0%) | 0 (0.0%) | 0 (0.0%) |
| Pain | 0 (0.0%) | 0 (0.0%) | 0 (0.0%) | 0 (0.0%) |
| Induration | 0 (0.0%) | 0 (0.0%) | 0 (0.0%) | 0 (0.0%) |
| Swelling | 0 (0.0%) | 0 (0.0%) | 0 (0.0%) | 0 (0.0%) |
| Erythema | 0 (0.0%) | 0 (0.0%) | 0 (0.0%) | 0 (0.0%) |
| Pruritus | 0 (0.0%) | 0 (0.0%) | 0 (0.0%) | 0 (0.0%) |
| **Systematic adverse reactions** | |  |  |  |
| Any (%) | 1 (2.5%) | 0 (0.0%) | 1 (2.5%) | 0 (0.0%) |
| Grade 2 | 1 (2.5%) | 0 (0.0%) | 1 (2.5%) | 0 (0.0%) |
| Fever | 0 (0.0%) | 0 (0.0%) | 0 (0.0%) | 0 (0.0%) |
| Fatigue | 1 (2.5%) | 0 (0.0%) | 1 (2.5%) | 0 (0.0%) |
| Grade 2 | 1 (2.5%) | 0 (0.0%) | 1 (2.5%) | 0 (0.0%) |
| Myalgia | 0 (0.0%) | 0 (0.0%) | 0 (0.0%) | 0 (0.0%) |
| Vertigo | 0 (0.0%) | 0 (0.0%) | 0 (0.0%) | 0 (0.0%) |
| Anorexia | 0 (0.0%) | 0 (0.0%) | 0 (0.0%) | 0 (0.0%) |
| Rash | 0 (0.0%) | 0 (0.0%) | 0 (0.0%) | 0 (0.0%) |
| Cough | 0 (0.0%) | 0 (0.0%) | 0 (0.0%) | 0 (0.0%) |
| Arthralgia | 0 (0.0%) | 0 (0.0%) | 0 (0.0%) | 0 (0.0%) |
| Dyspnea | 0 (0.0%) | 0 (0.0%) | 0 (0.0%) | 0 (0.0%) |
| Nausea | 0 (0.0%) | 0 (0.0%) | 0 (0.0%) | 0 (0.0%) |
| Pharyngalgia | 0 (0.0%) | 0 (0.0%) | 0 (0.0%) | 0 (0.0%) |
| Syncope | 0 (0.0%) | 0 (0.0%) | 0 (0.0%) | 0 (0.0%) |
| Vomiting | 0 (0.0%) | 0 (0.0%) | 0 (0.0%) | 0 (0.0%) |
| **Unsolicited adverse reactions** | |  |  |  |
| **Injection site adverse reactions** | |  |  |  |
| Any (%) | 0 (0.0%) | 0 (0.0%) | 0 (0.0%) | 0 (0.0%) |
| **Systematic adverse reactions** | |  |  |  |
| Any (%) | 0 (0.0%) | 0 (0.0%) | 0 (0.0%) | 0 (0.0%) |

**Supplementary Figure 1. The baseline antibody response and IFN-γ SFU/ million PBMCs 4-8 months after second dose**

1. Baseline IFN-γ SFU/ million PBMCs 4-8 months after second dose
2. Baseline antibody response 4-8 months after second dose evaluated by pVNT.
3. Baseline antibody response 4-8 months after second dose evaluated by sVNT，anti-RBD antibody，and anti-RBD IgG.

**Supplementary Figure 2.** Humoral immune responses after the third-dose vaccination evaluated by sVNT(IU/mL) .


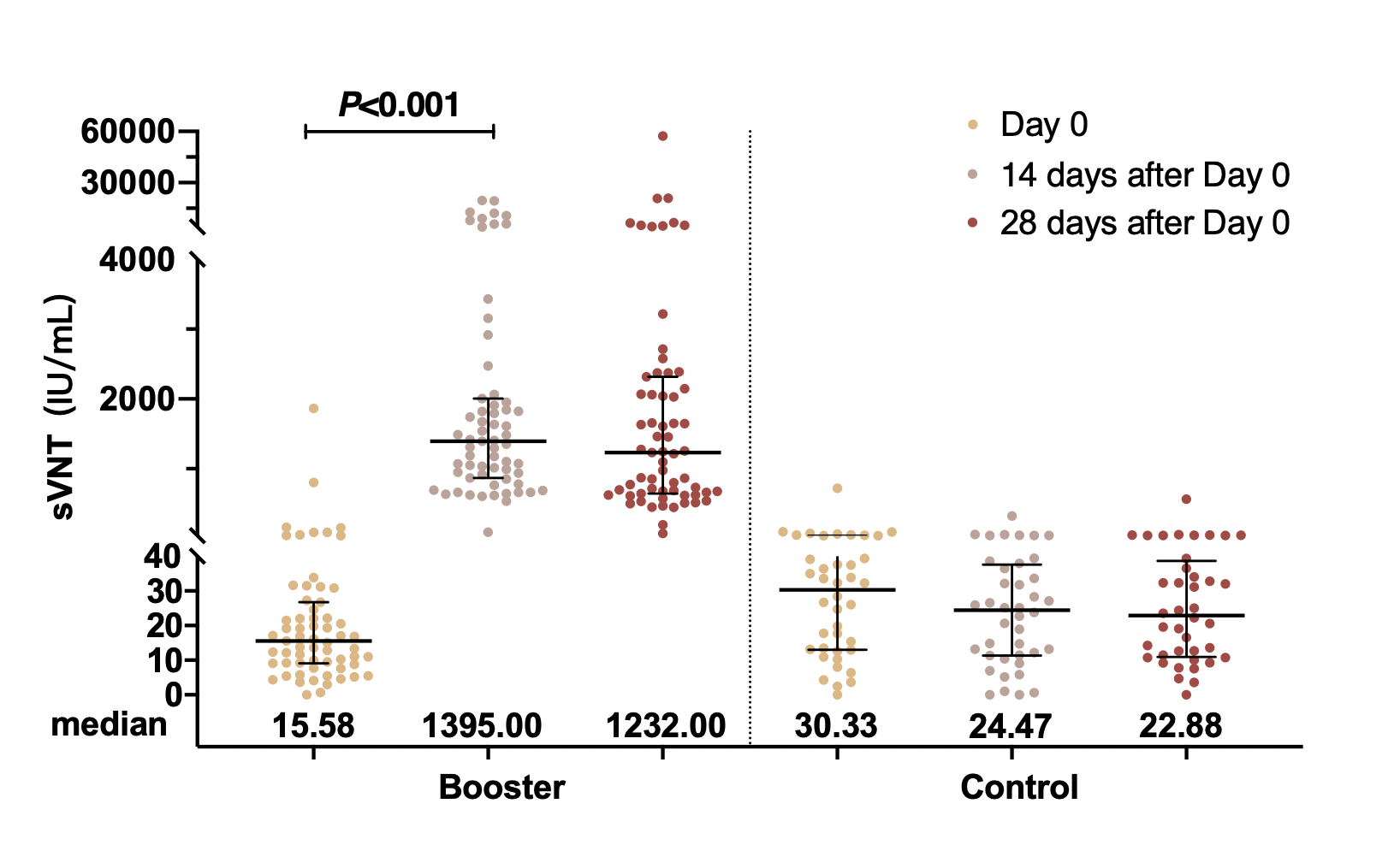


**Supplementary Figure 3. Subgroup analysis of antibody response in boost group evaluated by age (<40 yo, and≥40 yo) and gender.**

1. Subgroup analysis of antibody response in boost group evaluated by gender
2. Subgroup analysis of antibody response in boost group evaluated by age (<40 yo, and≥40 yo)

**Supplementary Figure 4. Subgroup analysis of T-cell response in boost group evaluated by age and gender.**

1. Subgroup analysis of a T-cell response in boost group evaluated by gender
2. Subgroup analysis of T-cell response in boost group evaluated by age (<40 yo, and≥40 yo)
